# Bioinspired DNase‐I‐Coated Melanin‐Like Nanospheres for Modulation of Infection‐Associated NETosis Dysregulation

**DOI:** 10.1002/advs.202001940

**Published:** 2020-10-20

**Authors:** Hee Ho Park, Wooram Park, Yun Young Lee, Hyelim Kim, Hee Seung Seo, Dong Wook Choi, Ho‐Keun Kwon, Dong Hee Na, Tae‐Hyung Kim, Young Bin Choy, June Hong Ahn, Wonhwa Lee, Chun Gwon Park

**Affiliations:** ^1^ Department of Biotechnology and Bioengineering Kangwon National University Chuncheon Gangwon‐do 24341 Republic of Korea; ^2^ Department of Biomedical‐Chemical Engineering The Catholic University of Korea Bucheon 14662 Republic of Korea; ^3^ Department of Biomedical Engineering Seoul National University College of Medicine Seoul 03080 Republic of Korea; ^4^ College of Pharmacy Chungnam National University Daejeon 34134 Republic of Korea; ^5^ Department of Biomedical Engineering SKKU Institute for Convergence Sungkyunkwan University (SKKU) Suwon 16419 Republic of Korea; ^6^ Department of Cancer Biology Dana‐Farber Cancer Institute Harvard Medical School Boston MA 02215 USA; ^7^ Department of Microbiology and Immunology Yonsei University College of Medicine Seoul 03722 Republic of Korea; ^8^ College of Pharmacy Chung‐Ang University Seoul 06974 Republic of Korea; ^9^ School of Integrative Engineering Chung‐Ang University Seoul 06974 Republic of Korea; ^10^ Division of Pulmonology and Allergy Department of Internal Medicine College of Medicine Yeungnam University and Regional Center for Respiratory Diseases Yeungnam University Medical Center Daegu 42415 Republic of Korea; ^11^ Aging Research Center Korea Research Institute of Bioscience and Biotechnology (KRIBB) Daejeon 34141 Republic of Korea; ^12^ Department of Biomedical Engineering SKKU Institute for Convergence Sungkyunkwan University (SKKU) Suwon Republic of Korea; ^13^ Biomedical Institute for Convergence at SKKU (BICS) Sungkyunkwan University 2066 Seobu‐ro, Jangan‐gu Suwon 16419 Republic of Korea; ^14^ Center for Neuroscience Imaging Research Institute for Basic Science (IBS) Suwon 16419 Republic of Korea; ^15^ Department of Intelligent Precision Healthcare Convergence SKKU Institute for Convergence Sungkyunkwan University (SKKU) Suwon 16419 Republic of Korea

**Keywords:** Bioinspiration, COVID‐19, DNase‐I, Nanospheres, NETosis

## Abstract

The current outbreak of the beta‐coronavirus (beta‐Cov) severe acute respiratory syndrome coronavirus 2 (SARS‐CoV‐2) began in December 2019. No specific antiviral treatments or vaccines are currently available. A recent study has reported that coronavirus disease 2019 (COVID‐19), the disease caused by SARS‐CoV‐2 infection, is associated with neutrophil‐specific plasma membrane rupture, and release excessive neutrophil extracellular traps (NETs) and extracellular DNAs (eDNAs). This mechanism involves the activation of NETosis, a neutrophil‐specific programmed cell death, which is believed to play a crucial role in COVID‐19 pathogenesis. Further progression of the disease can cause uncontrolled inflammation, leading to the initiation of cytokine storms, acute respiratory distress syndrome (ARDS), and sepsis. Herein, it is reported that DNase‐I‐coated melanin‐like nanospheres (DNase‐I pMNSs) mitigate sepsis‐associated NETosis dysregulation, thereby preventing further progression of the disease. Recombinant DNase‐I and poly(ethylene glycol) (PEG) are used as coatings to promote the lengthy circulation and dissolution of NET structure. The data indicate that the application of bioinspired DNase‐I pMNSs reduce neutrophil counts and NETosis‐related factors in the plasma of SARS‐CoV‐2 sepsis patients, alleviates systemic inflammation, and attenuates mortality in a septic mouse model. Altogether, the findings suggest that these nanoparticles have potential applications in the treatment of SARS‐CoV‐2‐related illnesses and other beta‐CoV‐related diseases.

## Graphical abstract

It is reported here that DNase‐I‐coated melanin‐like nanospheres (DNase‐I pMNSs) are able to modulate COVID‐19‐associated NETosis dysregulation and prevent further progression of the disease. The DNase‐I pMNSs have potential applications in the treatment of SARS‐CoV‐2‐related illnesses and other beta‐CoV‐related diseases.

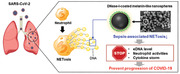

## 1 Introduction

In recent decades, there have been recurrence of large‐scale epidemics attributed to coronaviruses such as severe acute respiratory syndrome coronavirus (SARS‐CoV), Middle East respiratory syndrome coronavirus (MERS‐CoV), and most recently, SARS‐CoV‐2, which was discovered in patients with severe pneumonia in December, 2019.^[^ ^1^, ^2^, ^3^ ^]^ CoVs are classified into four categories, that is, alpha‐CoV, beta‐CoV, gamma‐CoV, and delta‐CoV. Interestingly, SARS‐CoV, MERS‐CoV, and SARS‐CoV‐2 are beta‐CoV lineage viruses. Currently, there are no specific antiviral treatments or vaccines available for these beta‐CoVs; therefore, it is crucial to develop novel treatment strategies to address the rapid spread of this virus.

The full spectrum of coronavirus disease 2019 (COVID‐19)—the disease associated with SARS‐CoV‐2 infection—ranges from mild respiratory tract illness to severe progressive pneumonia, acute respiratory distress syndrome (ARDS), serum pro‐inflammatory cytokine elevation (cytokine storm), multiple organ failure (MOF), and death.^[^ ^1^ ^]^ SARS‐CoV‐2 infections are associated with both uncontrolled inflammatory innate immune responses and impaired adaptive immune responses, leading to local and systemic tissue damage.^[^ ^4^ ^]^ The late stage of the disease is difficult to treat, as the radical immune reactions propagate toward cytokine storms, acute respiratory distress syndrome (ARDS), and septic shock, leading to increased patient mortality.^[^ ^5^, ^6^ ^]^ The underlying mechanism as well as initiators that trigger and propagate the cytokine storm, subsequently leading to the development of severe COVID‐19 are unknown.^[^ ^4^ ^]^ A recent study has proposed that neutrophilia might be linked to poor outcomes in patients with severe COVID‐19,^[^ ^5^ ^]^ and it is believed to play a critical role in COVID‐19 pathogenesis.^[^ ^7^ ^]^ NETosis, a special type of neutrophil‐specific programmed cell death, is a process in which reticular structures consisting of chromatin and granular proteins are formed.^[^ ^8^, ^9^ ^]^ It has been suggested that excessive amount of NETosis, which lead to a hyperinflammatory response,^[^ ^10^ ^]^ is associated with organ damage and MOF.^[^ ^11^ ^]^ Hence, it is postulated that aberrant activation of neutrophils followed by release of neutrophil extracellular traps (NETs) and extracellular DNAs (eDNAs) in the peripheral blood may contribute to organ damage and mortality in sepsis‐related diseases.^[^ ^12^ ^]^ It is predicted that similar outcomes may arise in COVID‐19 patients.

Inspired by our observations and based on a recently published report,^[^ ^7^ ^]^ we suggest that the dissolution of a basic constituent of NET structure—DNA—using DNase‐I may be an appropriate strategy for preventing NET‐related pathogenesis in SARS‐CoV‐2 patients. Previously, it had been demonstrated that the delivery of a recombinant DNase‐I by inhalation led to the dissolution of NETs in the airways of cystic fibrosis (CF) patients, that resulted in clear mucus and ameliorated symptoms.^[^ ^13^ ^]^ In addition, the use of an actin‐resistant DNase in CF patients in Phase I and II clinical trials showed promising results (NCT02605590, NCT02722122). When tested using animal models, delivery of DNase‐I through the airways resulted in increased survival,^[^ ^14^, ^15^, ^16^ ^]^ leading us to believe that DNase‐I may help dissolve NETs and prevent further progression to ARDS and sepsis in severe COVID‐19 patients. However, exogenously administered recombinant DNase‐I showed only a modest effect, presumably due to the short half‐life of DNase‐I in the blood plasma. Previous studies have reported that conjugation of DNase‐I onto the surface of nanoparticles enhances the stability and preserves the activity of DNase‐I in blood plasma.^[^ ^17^, ^18^ ^]^

In this study, we report for the first time, demonstration of DNase‐I‐coated melanin‐like nanospheres (DNase‐I pMNSs) for modulation of sepsis‐associated NETosis dysregulation. Polydopamine‐based DNase‐I MNSs have a structure similar to that of squid ink and skin melanin.^[^ ^19^, ^20^, ^21^, ^22^ ^]^ DNase‐I was readily attached to the nanoparticle surface through the excellent adhesive properties of polydopamine.^[^ ^23^, ^24^ ^]^ Intravenous injection of bioinspired DNase‐I MNSs alleviated systemic inflammation and attenuated mortality in a septic mouse model. We also show that exogenously administered DNase‐I MNSs exhibit a suppressive effect against neutrophil activities, eDNA level, and cytokine storm. Thus, our data support further investigation of DNase‐I MNSs as potential tools to treat SARS‐CoV‐2‐mediated illnesses.

## 2 Results

### 2.1 Increased Neutrophil and Reduced Lymphocyte Counts in Severe COVID‐19 Patients

Computed tomography (CT) was performed, and blood samples from COVID‐19 patients were analyzed on their admission to the hospital (Figure S1, Supporting Information, and **Table** **1**). CT imaging revealed that patients with severe clinical manifestations had severe lung tissue damage, indicating an increase in the severity of septic symptoms (Figure S1, Supporting Information). As reported previously, severe clinical manifestations such as ARDS and sepsis, were mostly observed in elderly patients (mean age 72.2 ± 13.1) who showed increased neutrophil counts and decreased lymphocyte counts when compared with the less severe group (Table 1).^[^ ^25^, ^26^ ^]^ The later symptom lymphopenia is widely recognized to be associated with the severity of diseases.^[^ ^22^ ^]^

**Table 1:** Laboratory findings of COVID‐19 patients at the time of admission to hospital

	All patients (*n* = 60)	Mild patients (*n* = 40)	Severe patients (*n* = 20)	*p*‐Value
**Characteristics**				
Age [y]	33.7 ± 16.4	55.4 ± 17.1	72.2 ± 13.1	0.014
ARDS	11 (18.3)	0 (0)	11 (55)	<0.001
Sepsis	13 (21.7)	0 (0)	13 (65)	<0.001
Discharged	40	40 (100)	0 (0)	<0.001
Died	16	0 (0)	16 (80%)	<0.001
**Complete blood count**				
White blood cell count, × 10^9^ L^−1^ [normal range 4–10]	6.5 ± 3.4	5.9 ± 3.6	9.1 ± 2.6	0.005
Neutrophil count, × 10^9^ L^−1^ [normal range 3–7]	4.6 ± 3.4	4.1 ± 3.2	7.7 ± 3.3	<0.001
Lymphocyte count, × 10^9^ L^−1^ [normal range 1.5–4]	1.4 ± 0.7	1.5 ± 0.7	0.8 ± 0.3	<0.001
Hemoglobin, g dL^−1^ [normal range 12–17]	13.0 ± 1.6	13.5 ± 1.7	13.0 ± 1.6	0.253
Platelets, × 10^9^ L^−1^ [normal range 140–400]	237.3 ± 104.9	245.0 ± 107.9	186.7 ± 64.9	0.061

Data are presented as mean ± SD (range) or number (percentage). Mild patients: non‐ICU, no symptom; severe patients: ICU, ventilator, oxygen treatment, ARDS, and sepsis.

### 2.2 Increased Levels of NETosis Markers in Severe COVID‐19 Patients

Levels of extracellular DNA (eDNA), DNase‐I and citrullinated histone H3 (Cit‐His H3) were measured in 20 healthy control volunteers (normal group), and 60 blood samples from COVID‐19 patients, including mild patients and severe patients. The median serum eDNA level in the normal group was 0.41 (0.29–0.53) µg mL^−1^. In comparison, the level of eDNA was slightly increased to 0.85 (0.58–1.12 µg mL^−1^) in the 40 mild COVID‐19 patients and drastically increased to 2.83 (2.46–3.20) µg mL^−1^ in the 20 severe COVID‐19 patients (**Table** **2**). The median serum Cit‐His H3 levels in the blood samples showed much more drastic differences. In the normal group, the Cit‐His H3 median level was slightly increased from 0.05 (0.04–0.06) µg mL^−1^ to 0.30 (0.02–0.62) µg mL^−1^ in mild COVID‐19 patients, and significantly increased to 17.74 (14.83–20.65) µg mL^−1^ in severe COVID‐19 patients. A slightly different effect was observed for DNase‐I, that is, in the normal group, the median DNase‐I level was slightly increased from 2.11 (1.73–2.49) µg mL^−1^ to 3.11 (2.04–4.18) µg mL^−1^ in the mild COVID‐19 patients, but decreased to 0.97 (0.67–1.27) µg mL^−1^ in severe COVID‐19 patients.

**Table 2:** NETosis markers in COVID‐19 patients

	Normal patients (*n* = 20)	Mild patients (*n* = 40)	Severe patients (*n* = 20)
**NETosis**			
eDNA [mg mL^−1^]	0.41 ± 0.12	0.85 ± 0.27	2.83 ± 0.37***
DNase‐I [mg mL^−1^]	2.11 ± 0.38	3.11 ± 1.07	0.97 ± 0.30***
Cit‐His H3 [mg mL^−1^]	0.05 ± 0.01	0.30 ± 0.32	17.74 ± 2.91***

Statistical analysis was performed using a two‐tailed unpaired *t*‐test. Data are presented as mean ± SEM. ^***^ *p* < 0.001. eDNA, extracellular DNA; Cit‐His H3, citrullinated histone H3.

### 2.3 Preparation and Characterization of DNase‐I pMNSs

**Figure 1:**
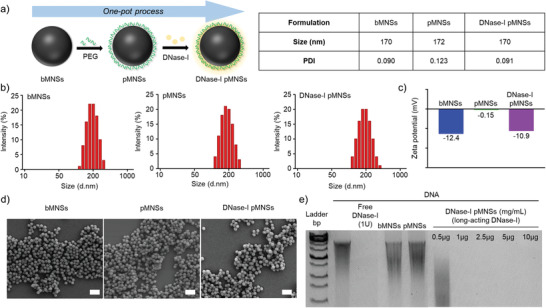
Physicochemical characterization of pMNSs. a) Preparation of DNase‐I pMNSs. b) Size distribution of bMNSs, pMNSs, and DNase‐I pMNSs. c) Zeta potentials of bMNSs, pMNSs, and DNase‐I pMNSs. d) Scanning electron microscopy (SEM) images of bMNSs, pMNSs, and DNase‐I pMNSs (scale bar: 500 nm). e) Migration profile of pure DNA after digestion with free DNase‐I, pMNSs, and bMNSs, as well as various amounts of DNase‐I pMNSs. pMNSs, PEG‐coated melanin‐like nanospheres; bMNSs, bare bioinspired melanin‐like nanospheres.

The bare bioinspired melanin‐like nanospheres (bMNSs) were surface‐modified with DNase‐I to improve the stability and thus activity of DNase‐I (**Figure** **1**a).^[^ ^27^ ^]^ The melanin‐like nanoparticles were synthesized through oxidation of dopamine based on the protocol published in our previous paper.^[^ ^21^ ^]^ Then, DNase‐I and PEG were immobilized on the surface of nanoparticles in a one‐pot process. The particle sizes of bMNSs, pMNSs, and DNase‐I pMNSs were similar, approximately 170 nm (Figure 1b). The surface charge of bMNSs was measured to be −12.4 mV, which neutralized to −0.15 mV when the PEG coating was introduced to generate pMNSs (Figure 1c). The surface of the DNase‐I pMNSs was negatively charged (−10.9 mV) due to coating with DNase‐I. Data also confirmed the spherical appearance and relatively monodispersed size of the nanoformulations (Figure 1d). The DNA degradation capability of DNase‐I pMNSs was evaluated using agarose gel electrophoresis (Figure 1e). Free DNase‐I (1 U) completely degraded 1 µg DNA. pMNSs and bMNSs did not degrade DNA due to the absence of DNase‐I on their surface. However, DNase‐I pMNSs degraded DNA at a concentration > 1.0 µg of nanospheres, suggesting that the effect was due to the activity of coated DNase‐I on pMNSs. To evaluate the amount of DNase‐I that can be bound to pMNSs, nanoparticles were prepared with various ratios of pMNSs and DNase‐I. The binding contents and binding efficiency of DNase‐I on pMNSs were measured through BCA assay (Table S1, Supporting Information). As the feed amount of DNase‐I increased, the actual amount of DNase‐I binding to the nanoparticles increased, but the binding efficiency of DNase‐I was limited to less than 83%. However, the DNase‐I pMNSs3 containing ≈45% of DNase‐I showed excellent stability and DNA degradation properties, thus DNase‐I pMNSs3 was selected and used in further experiments. Then, to analyze the binding stability of nanoparticles and DNase‐I, the activity of DNase‐I over time in PBS containing 10% FBS or PBS only media, was tested. When DNase activity was tested after incubation of long‐acting DNase‐I in media containing 10% FBS for 72 h (Figure S2(a), Supporting Information), the activity of DNase‐I pMNSs was maintained for up to 36 h, but the activity decreased after 48 h. However, in PBS only media (Figure S2(b), Supporting Information), the activity of DNase‐I pMNSs was maintained for up to 72 h.

### 2.4 Exogenous DNase‐I pMNSs Alleviated NETosis Factors in the Plasma and Neutrophils of Severe COVID‐19 Patients

**Figure 2:**
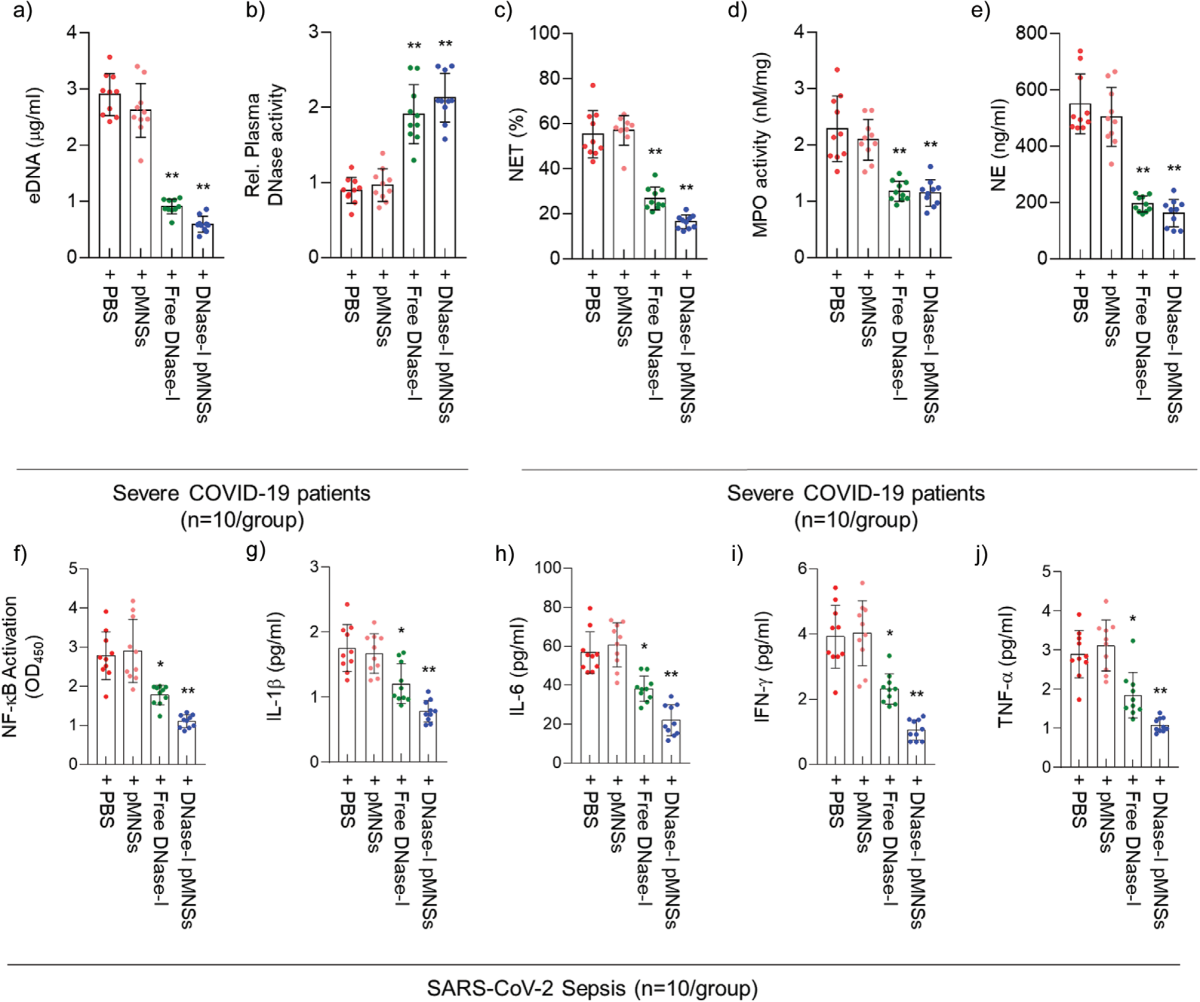
DNase‐I pMNSs suppress NETosis factors and cytokine storm via down‐regulation of NF‐*κ*B activation. a) eDNA and b) DNase‐I levels in the plasma of severe COVID‐19 patients, as well as c) NET, d) MPO, and e) NE levels in neutrophils isolated from severe COVID‐19 patients. Plasma and neutrophil samples were collected from 20 severe COVID‐19 patients. f) Suppression of NF‐*κ*B activation in severe COVID‐19 patients’ PBMCs after the treatment of free‐DNase‐I or DNase‐I pMNSs. g–j) Reduction of secreted cytokine levels, including g) IL‐1*β*, h) IL‐6, i) IFN‐*γ*, and j) TNF‐*α* in severe COVID‐19 patients’ PBMCs after the treatment of free‐DNase‐I or DNase‐I pMNSs. Statistical analysis was performed using a two‐tailed unpaired *t*‐test. Data are presented as mean ± SEM. ^*^ *p* < 0.05, ^**^ *p* < 0.01. pMNSs, PEG‐coated melanin‐like nanospheres; eDNA: Extracellular DNAs; NET, Neutrophil extracellular trap; MPO, Myeloperoxidase; NE, Neutrophil elastase; PBMC, Peripheral blood mononuclear cells; NF‐kB, Nuclear factor kappa‐light‐chain‐enhancer of activated B cells; IL‐1*β*, Interleukin‐1 beta; IL‐6, Interleukin‐6; IFN‐ *γ*, Interferon‐*γ*; TNF‐*α*, tumor necrosis factor‐*α*.

After observing an increase in the levels of NETosis‐related factors, we hypothesized the necessity to compensate for the loss of endogenous DNase‐I in severe COVID‐19 patients. We therefore validated the effects of DNase‐I pMNSs on reduction of the neutrophils in NETosis. To demonstrate the effects of DNase‐I on DNA degradation, we treated the plasma of severe COVID‐19 patients with either free DNase‐I or DNase‐I pMNSs. The results showed that both forms of DNase‐I significantly reduced the eDNA levels (**Figure** **2**a), and that exposure of DNase‐I to the plasma of severe COVID‐19 patients increased the activity of DNase‐I (Figure 2b). We also observed markedly reduced NET levels, MPO activity, and NE levels in neutrophils of severe COVID‐19 patients upon treatment with DNase‐I pMNSs (Figure 2c–e).

As shown in Table 1, over 50% of the severe COVID‐19 patients were diagnosed with ARDS and sepsis on admission to the hospital. Typically, sepsis is accompanied by a cytokine storm,^[^ ^4^ ^]^ a deadly uncontrollable systemic inflammatory response resulting from the burst of vast amounts of pro‐inflammatory cytokines by immune effector cells.^[^ ^28^ ^]^ Therefore, we evaluated the effects of DNase‐I pMNSs on NF‐*κ*B activation and cytokine secretion from neutrophils. The results showed that the activity of NF‐*κ*B and secretion of cytokines IL‐1*β*, IL‐6, IFN‐*γ*, and TNF‐*α* were slightly reduced upon treatment with free DNase‐I, and were further drastically reduced upon treatment with DNase‐I pMNSs (Figure 2f–j).

### 2.5 DNase‐I pMNSs Alleviated NETosis Factors in a Septic Mouse Model

**Figure 3:**
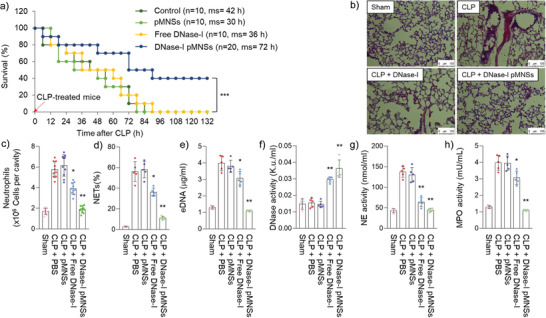
Anti‐NETosis effects of DNase‐I pMNSs in the sepsis mouse model. Male C57BL/6 mice (*n* = 60/group pooled from 3 independent experiments) were administered PBS, PEG‐Nano (100 units), free DNase‐I (100 units), or DNase‐I pMNSs (100 units) 12 h and 24 h after CLP, and the blood was withdrawn and measured 24 h later (48 h post‐CLP‐operation). a) Kaplan‐Meier curves of CLP mice administered PBS, PEG‐Nano (100 units), free DNase‐I (100 units), or DNase‐I pMNSs (100 units). b) Histological changes in the lung 3 d after CLP treatment. Representative images from each group are shown (*n* = 5). Scale bar, 100 µm. c) Neutrophils, d) NETs, e) eDNA, f) DNase‐I activity, g) NE activity, and h) MPO activity were measured. Representative data from each group are shown (n = 5). The experiment was performed at least three times with replicates. Statistical analysis was performed using a two‐tailed unpaired *t*‐test. ^*^ *p* < 0.05, ^**^ *p* < 0.01. pMNSs: PEG‐coated melanin‐like nanospheres; CLP, Cecal ligation and perforation; PBS, Phosphate buffered saline; NETs, Neutrophil extracellular traps; eDNA: Extracellular DNAs; NE, Neutrophil elastase; MPO, Myeloperoxidase.

After confirming reduction in neutrophil counts and neutrophil‐related NETosis factors in severe COVID‐19 patients upon treatment with DNase‐I MNSs in vitro, we further validated the effects of DNase‐I pMNSs in vivo. Based on a recent report that neutrophil activity is a valid target for preventing or reducing sepsis and enhancing survival,^[^ ^7^ ^]^ we tested the exogenous administration of the DNase‐I pMNSs in an in vivo setting: cecal ligation and perforation (CLP)‐treated septic mouse model (**Figure** **3**a). CLP mouse model is the most frequently used model to investigate the complex molecular mechanisms of sepsis.^[^ ^29^ ^]^ Sepsis is induced via polymicrobial infectious focus within the abdominal cavity, followed by bacterial translocation into the blood compartment, which triggers a systemic inflammatory response. Similar phenotypes are observed in the CLP model, and severe COVID‐19 patients; NETosis, cytokine release syndrome (CRS), and multiple organ failure (MOF) syndrome can be induced in the CLP model. Hence, the CLP model can be used to represent the SARS‐CoV‐2‐induced severe COVID‐19 patients. The CLP model was considered an appropriate in vivo model for the current setting due to the absence of available alternatives. To confirm the anti‐NETosis effects of DNase‐I pMNSs, CLP‐operated mice were intravenously injected with phosphate‐buffered saline (PBS; control group), PEG‐Nano, free DNase‐I (100 units), or DNase‐I pMNSs (100 units) at 12 h and 24 h post‐CLP‐operation. We measured the DNase‐I activity 24 h after drug administration (48 h post‐CLP‐operation); Colón et al. measured the activity after 18 h,^[^ ^30^ ^]^ Yoshikawa et al. measured the activity after 24 and 48 h NET,^[^ ^31^ ^]^ and Czaikoski et al. measured the activity at 48 h.^[^ ^11^ ^]^ Previously, it was reported that neutrophil activity increases after CLP surgery as a function of time.^[^ ^32^ ^]^ To effectively suppress the rapidly increasing NET, the drug was administered at 12 and 24 h and the effect was confirmed 24 h after drug administration (48 h post‐CLP‐operation). All of the CLP‐operated mice died within 90 h of CLP induction when PBS, PEG‐Nano, or free DNase‐I were administered. This demonstrated the high mortality rate of the CLP‐operated sepsis model. Previously, the pharmacodynamics of recombinant human DNase‐I in the serum was analyzed, indicating a short half‐life,^[^ ^33^ ^]^ and a lack of in vivo effect of free DNase‐I. Interestingly, DNase‐I pMNSs demonstrated a 40% survival rate for the CLP‐operated septic mice for over 132 h, leading to the full recovery of these mice. We then confirmed the effect of DNase‐I pMNSs on the lungs, and we found a significant reduction in the morphological changes caused by CLP, including pulmonary edema, hemorrhage, alveolar collapse, and inflammatory cell infiltration (Figure 3b).

To further evaluate the efficacy of DNase‐I pMNS treatment in a therapeutic setting, we evaluated the levels of neutrophils, NETs, eDNA, DNase activity, NE activity, and MPO activity in the intraperitoneal cavity of CLP‐operated mice (Figure 3c–h). In agreement with the effects observed in the plasma and neutrophils of severe COVID‐19 patients (Figure 2), similar results were observed in which DNase‐I pMNSs induced a drastic reduction in the levels of peritoneal neutrophils, NETs, eDNA, NE activity, and MPO activity compared to those in the control group (PBS treatment) and free DNase‐I group. It was also clearly demonstrated that DNase activity increased dramatically in DNase‐I pMNSs group, compared to the control group (PBS treatment).

Next, we evaluated the effect of the DNase‐I pMNSs on the regulation of inflammatory responses in the CLP‐operated sepsis model. As mentioned before, systemic inflammation associated with sepsis often causes MOF syndrome,^[^ ^34^ ^]^ and it has been reported that the major organ targets of MOF syndrome are the kidney and liver.^[^ ^35^ ^]^ CLP treatment significantly increased the serum levels of hepatic injury markers alanine transferase (ALT) and aspartate transaminase (AST) (Figure S3a and b, Supporting Information), renal injury markers blood urea nitrogen (BUN) and creatinine (Figure S3c–d, Supporting Information), tissue injury marker lactate dehydrogenase (LDH) (Figure S3e, Supporting Information), and pneumonia and sepsis marker C‐reactive protein (CRP) (Figure S3f, Supporting Information). Treatment with DNase‐I pMNSs led to apparent reductions in the serum levels of all these markers.

**Figure 4:**
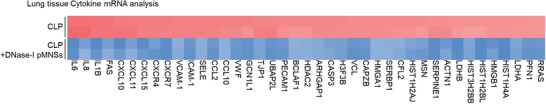
Cytokine levels in the lung tissue in CLP‐operated mouse model. Heat map of inflammation‐related genes in the lung tissue after DNase‐I pMNSs treatment (*n* = 3). CLP, Cecal ligation and perforation; pMNSs: PEG‐coated melanin‐like nanospheres.

We also evaluated the absolute number of neutrophils, leukocytes, and total white blood cells (WBCs) in the blood of the CLP‐operated sepsis model (Figure S4a–c, Supporting Information). Similar to the effect shown in the intraperitoneal cavity of CLP‐operated sepsis model (Figure 3c), DNase‐I pMNSs caused a drastic reduction in the number of neutrophils and the total number of WBCs in the blood relative to those observed in the control group (PBS treatment) (Figure S4a and c, Supporting Information). However, compared to the control group (PBS treatment), no difference was observed in the number of leukocytes in the blood (Figure S4b, Supporting Information). Cytokine array analysis on lung tissue in the CLP‐operated mouse model revealed the upregulation of inflammatory cytokines (**Figure** **4**). The upregulated cytokines included IL‐6, IL‐8, IL‐1*β*, CCL2, etc. The high level of cytokines observed in the lung tissue in the CLP‐operated mouse model supports the sudden rise in the systemic inflammatory response, including the triggering of the “cytokine storm” after SARS‐CoV‐2 infection. However, upon treatment with DNase‐I pMNSs, the level of cytokines such as IL‐6, IL‐8, IL‐1*β*, and CCL2, was significantly reduced in the lung tissue samples. As SARS‐CoV‐2 causes damage to vascular endothelial cells and induces severe lung damage, we evaluated whether DNase‐I can protect vascular barrier disruption by observing the transendothelial permeability in the septic mouse model (Figure S5, Supporting Information). As a result, free DNase‐I was only effective initially and the effect disappeared after 72 h upon administration, but in the group administered with DNase‐I pMNSs, the survival rate was maintained even after 72 h. The observed features in the blood of animal models support the improved outcomes after the DNase‐I pMNSs administration. To verify whether the ability of the nanoparticle‐bound is DNase‐I concentration dependent, we generated various concentrations of DNase‐I pMNSs, and observed concentration‐dependent survival rate in vivo (i.e., DNase‐I as variable and nanoparticles as constant; 25, 50, and 100 units) (Figure S6, Supporting Information).

## 3 Discussion

NETosis, a neutrophil‐specific oxidative explosion‐dependent process, leads to the release of neutrophil extracellular traps (NETs).^[^ ^10^ ^]^ NETosis is a special type of programmed cell death in which reticular structures consisting of a DNA backbone and a number of functional proteins are formed. Initially, the neutrophil elastase (NE) degrades the core histone protein and the linker H1 histone protein, resulting in neutrophil nuclei losing their characteristic shape. Then, a combination of nuclear rupture and granular membranes leads to chromatin decondensation and the release of NETs into the extracellular space, enhanced by myeloperoxidase (MPO). These NETs correspond to extracellular filaments of uncondensed chromatin coated by numerous granular proteins. During this process, Cit‐His H3 is thought to be involved in the formation of NETs. NETs have been linked to severe infections such as sepsis, in which the release of chromatin with neutrophil granular proteins serves as an additional defense of the innate immune system against circulating microbes, including bacteria, fungi, protozoa, and viruses. Recently, Zuo et al. verified that sera from patients with COVID‐19 have elevated levels of cell‐free DNA (eDNA), myeloperoxidase‐DNA (MPO‐DNA), and citrullinated histone H3 (cit‐His H3), and demonstrated that the latter two (MPO and cit‐His H3) are specific markers of NETs.^[^ ^36^ ^]^ In patients with severe COVID‐19, lymphopenia has been observed as a collective and representative characteristic, accompanied by an increased ratio of neutrophils as well as an increase in the absolute neutrophil count.^[^ ^25^, ^26^ ^]^ In addition, a recent study has proposed that NETosis is perhaps linked to poor outcomes in patients with COVID‐19 and is believed to play a crucial role in COVID‐19 pathogenesis.^[^ ^7^ ^]^ It is postulated that aberrant activation of neutrophils, an increase in the release of NETs, and eDNAs in peripheral blood may contribute to organ damage and mortality in COVID‐19 patients.

A recently published report^[^ ^7^ ^]^ and in our own independent work have demonstrated elevated levels of NETosis markers, such as eDNA, MPO and NE.^[^ ^10^, ^37^, ^38^ ^]^ These are characteristic features of critically ill patients with COVID‐19.^[^ ^39^ ^]^ Hence, we hypothesized that DNase‐I could be used to ameliorate the dissolution of the main constituent of NET structure, DNA, and thus prevent NET‐related pathogenesis, including progression toward ARDS and sepsis in severe COVID‐19 patients. However, it is quite challenging to treat patients with high levels of NETosis, and it is linked with high mortality and morbidity rates. Therefore, a novel NETosis‐targeting agent able to tackle and reduce NETosis in the blood is essential to prevent further progression toward sepsis. Currently, there are no FDA‐approved drugs to treat severe COVID‐19. Existing antivirals and antimalarials that are being used in clinical trials for the treatment of SARS‐CoV‐2 have shown therapeutic efficacy in only patients with mild conditions and not in severe COVID‐19 patients with ARDS or sepsis. Remdesivir has shown some promising results in mild COVID‐19 patients; however, it is still an investigational antiviral drug and is not currently FDA‐approved to treat or prevent any diseases, including COVID‐19. This highlights the urgent need for an alternative means of treating severe COVID‐19 patients by effectively suppressing the inflammation‐mediated MOF syndrome.

Although degrading the NET structure could serve as a promising strategy to suppress NET‐related complications in COVID‐19 patients, it has been reported that the half‐life of DNase‐I in the blood is very short.^[^ ^33^ ^]^ Previously, a polydopamine‐coated polymeric nanoparticle was proposed as a long‐acting DNase‐I formulation.^[^ ^17^, ^18^ ^]^ In this study we report, we demonstrated DNase‐I‐coated melanin‐like nanospheres (DNase‐I pMNSs) for the amelioration of sepsis‐associated NETosis (Figure 1). The DNase‐I pMNSs possess a structure similar to that of squid ink and skin melanin.^[^ ^19^, ^20^, ^21^, ^22^ ^]^ As melanin nanoparticles using polydopamine are processed in water without using organic solvents, the synthesis of melanin nanoparticles is environmentally friendly. In addition, the excellent adhesion properties of polydopamine enable the effective immobilization of the biomolecules on the surface of nanoparticles, thereby enabling various bioapplications. The interaction between the surface of melanin nanoparticles and DNase‐I is Michael addition or Schiff base reaction between the catechol group of melanin and the amine group of DNase‐I.^[^ ^40^, ^41^ ^]^ We observed that the DNase‐I pMNSs maintained their activity in a biologically relevant solution (media containing FBS) for 36 h (Figure S2, Supporting Information). This is thought to be because the DNase‐I attached on the nanoparticle surface could leech off or exchange in the presence of FBS. Nonetheless, it is assumed that the 36 h‐stability of the nanoparticles is sufficient even under the biological settings (during circulation in the blood containing human serum albumin) after injection.

We also performed a comparative study using blood samples from severe COVID‐19 patients and demonstrated the suppression of NETosis factors such as eDNA, NET, MPO, NE, and cytokine levels (Figure 2). Through the administration of exogenous DNase‐I pMNSs in the CLP‐operated septic mouse model, we demonstrated that DNase‐I pMNSs effectively reduce sepsis‐associated NETosis factors (Figures 3 and 4). We also showed that administration of the DNase‐I pMNSs significantly reduces NETosis factors such as eDNA, NET, MPO, and NE in the CLP‐operated septic mice (Figure 3). These observations are also consistent with cytokine array analysis in lung tissue from the CLP‐operated mouse model, which revealed that DNase‐I pMNS treatment lead to a reduction in the level of sepsis‐related pro‐inflammatory cytokines, such as IL‐6, IL‐8, IL‐1*β*, and CCL2 (Figure 4). In particular, intravenous injection of DNase‐I pMNSs alleviated the systemic inflammation and attenuated mortality in a septic mouse model, resulting in a 40% survival rate for the CLP‐operated septic mice at 132 h post‐induction (Figure 3a), demonstrating that the mice had fully recovered. The free DNase‐I is effective at the beginning of administration (Figure 3f), but in the group administered with DNase‐I pMNSs, it was confirmed that the effect lasted until 72 h and improved the survival rate (Figure 3a). The free DNase‐I is effective up to about 24 h after administration, and the DNase‐I‐bound nanoparticles (DNase‐I pMNSs) acted longer and improved the mortality (Figure 3a).

These results indicate that early administration of the DNase‐I pMNSs during the early phase of sepsis may slow progression toward ARDS and sepsis, thereby potentially treating the symptoms in severe COVID‐19 patients. Our data support further investigation of DNase‐I pMNSs for the treatment of severe COVID‐19‐mediated illnesses. Although the suggestion to use DNase‐I pMNSs as a potential treatment for severe COVID‐19 is based entirely on in vitro blood samples derived from severe COVID‐19 patients and in vivo studies using the CLP‐operated septic mouse model as a surrogate for SARS‐CoV‐2 infection, our results demonstrate that NETosis factor, cytokine release, and lung damage were significantly alleviated after treatment. In addition, sepsis‐associated inflammatory response including, NF‐*κ*B activation were inhibited upon the administration of DNase‐I pMNSs. It is worth noting that the efficacy of DNase‐I pMNSs needs to be further validated in an appropriate in vivo setting using an ARDS‐ or sepsis‐inducing infection animal model before moving forward to clinical trials on patients with COVID‐19.

## 4 Conclusion

Based on our data from severe COVID‐19 patients, we demonstrated that eDNA, a NETosis factor, is a potential target for the treatment of SARS‐CoV‐2‐induced sepsis. We suggest therapeutic delivery of a DNase‐I pMNSs for the suppression of eDNA, thereby alleviating the progression of sepsis in severe COVID‐19 patients. We confirmed the effectiveness of the DNase‐I pMNSs in blood samples collected from actual COVID‐19 patients as well as in a CLP‐operated septic mouse model. DNase‐I pMNSs is a promising bioinspired vehicle and demonstrates a potential new strategy for SARS‐CoV‐2‐induced sepsis therapy. There are currently no other reports that target the NETosis factor for the treatment of severe COVID‐19 patients. Recently, DNase‐I was delivered to dissolve NETs in patients with CF, and our findings implicate DNase‐I pMNSs as a suitable material for clinical translation and for preventing the progression of sepsis in severe COVID‐19 patients. Our findings indicate the possibility of using NETosis factors as diagnostic targets in severe COVID‐19 patients.

## 5 Experimental Section

##### Materials

Dopamine hydrochloride and poly(ethylene glycol) (PEG, four arm‐amine termini, HCl salt) were obtained from Sigma (PA, USA) and JenKem (TX, USA), respectively). NaOH solution (1 N) and Tris buffer (10 mM, pH 8.5) were purchased from Daejung (Suwon, Korea) and Biosesang (Seoul, Korea), respectively. Recombinant DNase‐I was purchased from Roche (Basel, Switzerland). Pierce BCA Protein assay kit was from Thermo Scientific (MA, USA). Dulbecco's phosphate‐buffered saline (PBS), and fetal bovine serum (FBS) were from Welgene (Gyeongsan, Korea) and Gibco (CA, USA), respectively.

##### Plasma Samples

At Yeungnam University Medical Center, whole blood samples were collected from patients after they were diagnosed with the SARS‐CoV‐2 infection at a public health center in Daegu, Republic of Korea. Patients with COVID‐19 sepsis were defined using criteria provided by the Sepsis Consensus Conference Committee.^[^ ^42^ ^]^ The human study protocol was approved by the Institutional Review Board of Yeungnam University Hospital at Daegu in Korea (YUH 2020‐03‐057, 2020‐05‐031‐001).

##### Neutrophil Counts of Patients with COVID‐19

Complete blood counts were analyzed in venous blood samples within 24 h of the admission of patients in the hospital. Neutrophil count was analyzed using a Sysmex XE‐2100 Automated Hematology System (TOA Medical Electronics, Kobe, Japan).

##### NET Enzyme‐Linked Immunosorbent Assay (ELISA)

NETs were generated from freshly isolated neutrophils (1  ×  10^5^ cells) by stimulating the cells with phorbol‐myristate acetate (25 nM) (PMA, Sigma‐Aldrich, MO, USA) or control media (RPMI 1640 supplemented with glutamine, penicillin, and streptomycin) and analyzed using a fluorometric technique as previously described.^[^ ^43^ ^]^ NET production was measured as arbitrary fluorescent units (AFUs).

##### MPO ELISA

Plasma samples were analyzed to quantify the release of granule matrix proteins upon degranulation in peripheral blood mononuclear cells (PBMCs) of SARS‐CoV‐2‐infected patients and mice, using a human MPO ELISA kit (BMS2038INST, Invitrogen) and mouse myeloperoxidase ELISA kit (MBS700747, MyBioSource), respectively.

##### Cit‐His H3 ELISA

Cit‐His H3 concentration in cell culture media or SARS‐CoV‐2 patient sera was determined using a human citrullinated histone H3 ELISA Kit (MBS7254090, MyBioSource).

##### Quantification of Plasma Extracellular DNA (eDNA)

To quantify eDNA from the plasma of SARS‐CoV‐2‐infected patients or mouse plasma, plasma samples were centrifuged at 16800 × *g* for 10 min and DNA was extracted using a Qiagen QIAamp DNA Mini Blood Mini Kit according to the manufacturer's protocols (Qiagen, Valencia, CA, USA) The purified eDNA was quantified using a NanoDrop spectrophotometer.

##### ELISA for DNase‐I Activity

Plasma samples were diluted 1:50 and analyzed using digestion buffer spiked with double‐stranded DNA (1 µg mL^−1^). Samples were stained with PicoGreen (Invitrogen) according to the manufacturer's protocol. After incubation at 37 °C for 5 h, reduction in PicoGreen staining (fluorescence emission, Em) was then measured using a fluorometer.

##### Preparation of DNase‐I pMNSs

Bare melanin‐like nanospheres (bMNSs) were synthesized by dopamine hydrochloride, as presented in our previous report^[^ ^21^ ^]^ (Figure 1). Dopamine hydrochloride (10 mg) was dissolved in deionized water (DW, 50 mL). Then, NaOH solution (50 µL, 1 N) was added to the dopamine hydrochloride solution. The reaction was stirred for 24 h, and the color gradually changed to black as the reaction proceeded. For purification, the prepared bMNSs were collected via centrifugation at 17 000 rpm (27237 × *g*‐force) for 20 min and washed with deionized water (DW) three times. To prepare DNase‐I pMNSs, surface engineering of bMNSs with DNase‐I was performed according to our previous reports.^[^ ^17^, ^18^ ^]^ The resulting bMNSs (10 mg) were re‐suspended in Tris buffer (5 mL, 10 mM, pH 8.5) containing DNase‐I (2, 5, 10, or 20 mg) and poly(ethylene glycol) (10 mg, PEG, four arm‐amine termini, HCL salt), and stirred at 4 °C for 3 h. The prepared DNase‐I pMNSs were purified in the same manner as above and washed with DW multiple times. We also prepared PEG‐coated MNSs (pMNSs) as a control, using the same method as for DNase‐I pMNSs, but without DNase‐I addition.

##### Characterization of MNS

The particle size and surface charge of nanospheres were measured by dynamic light scattering (DLS: Malvern Instruments, Southborough, Massachusetts). The morphology of the nanospheres was observed by field emission scanning electron microscopy (FE‐SEM: JEM‐7500F, Akishima, Japan). The enzymatic activity of DNase‐I pMNSs was confirmed via degradation of highly polymerized salmon sperm DNA (Sigma‐Aldrich, PA, USA) by gel electrophoresis.^[^ ^18^ ^]^ For this, bMNSs, pMNSs, or DNase‐I pMNSs were incubated with 1 µg of salmon sperm DNA for 10 min at 37 °C, followed by gel electrophoresis.

##### Neutrophil Isolation

For density gradient isolation of PBMCs, Percoll (pH 8.5–9.5; Sigma‐Aldrich, UK) was used as previously described.^[^ ^44^ ^]^ The PBMCs (95% purity and 97% viable according to trypan blue exclusion) were resuspended in RPMI 1640 media (Sigma‐Aldrich). For discontinuous density gradient centrifugation of neutrophils, 1‐step Polymorphs (Axis‐Shield, Oslo, Norway) were used. To further increase the purity, the neutrophil population was purified using CD45 antibody‐conjugated magnetic beads and magnetic‐activated cell sorting (MACS). Trypan blue dye exclusion showed that the general viability of the neutrophils was >95%. To analyze the effect of DNase‐I on inhibition of cytokine production, neutrophils were isolated from COVID‐19 patients and treated with DNase‐I MNSs. DNase‐I MNSs were also administrated to CLP‐operated septic mice. Supernatants were used for cytokine assay using ELISA, and cell lysates were used for analysis of NF‐kB activity.

##### NF‐*κ*B Activity

Nuclear extracts were prepared, and TransAM assays were performed as previously described.^[^ ^45^ ^]^ The activity of individual NF‐*κ*B subunits was determined via ELISA using NF‐*κ*B Family Transcription Factor Assay Kit (43296; Active Motif, Carlsbad, CA, USA). Briefly, nuclear extracts (2 µg) were placed into wells of NF‐*κ*B consensus oligonucleotide‐coated 96‐well plates. Plates were incubated with NF‐*κ*B primary antibody, and then binding was detected using HRP‐conjugated secondary antibody included with the kit. For analysis, the optical density (OD) at 450 nm was measured using a Tecan Spark microplate reader (Tecan, Austria GmbH, Austria).

##### Cytokine ELISAs

Levels of inflammatory cytokines IL‐1b, IL‐6, IFN‐g, and TNF‐*α* in the supernatant were measured after treating PBMCs with pMNSs, Free‐DNase‐I, and pMNSs‐DNase‐I (100 unit) using human ELISA kits (Quantikine ELISA, R&D Systems, Minneapolis, MN, USA) according to the manufacturer's protocols.

##### Animals

Animal experiments were carried out in accordance with protocols approved by the Institutional Animal Care and Use Committee (IACUC) of Sungkyunkwan University College of Medicine (IACUC No. No. SKKU IACUC2020‐06‐29‐2). Six‐ to seven‐week‐old C57BL/6 male mice (18–20 g) were obtained from Orient Bio (Seongnam, Korea). Mice were used after a 12‐day acclimatization period. Five animals per cage were housed under controlled temperature at 20–25 °C and humidity of 40–45% with a 12:12 h light/dark cycle. Mice were fed a normal rodent pellet diet and supplied with water ad libitum.

##### Cecal Ligation and Puncture

The CLP‐operated septic mouse model was prepared as previously described.^[^ ^29^ ^]^ Briefly, a 2‐cm midline incision was made to expose the cecum and adjoining intestine. The cecum was then ligated tightly using a 3.0‐silk suture 5.0 mm from the cecal tip, punctured with a 22‐gauge needle, and then gently squeezed to extrude feces from the perforation site. The cecum was then returned to the peritoneal cavity, and the laparotomy site was sutured using 4.0‐silk. For sham operations, the cecum of animals was surgically exposed, but not ligated or punctured, and then returned to the abdominal cavity.

##### In Vivo Neutrophil Migration Assays

To assess neutrophil migration, CLP‐operated mice were treated with pMNSs, free DNase‐I, and pMNSs‐DNase‐I (100 units) 6 h after CLP surgery. Mice were then euthanized, and the peritoneal cavities were washed with 5 mL of normal saline. Neutrophils were counted using an auto hematology analyzer (Mindray, BC‐5000 Vet). Results are expressed as neutrophils × 10^6^ per peritoneal cavity.

##### RNA Analysis

RNA from lung tissue was extracted using an RNeasy mini‐kit (Qiagen Venlo, Netherlands) according to the manufacturer's protocols. RNA‐seq libraries were prepared using the TruSeq RNA Sample Prep kit v2 (Illumina) according to the manufacturer's protocols. RNA‐seq libraries were pair‐end sequenced on an Illumina Hi‐seq 3000/4000 SBS kit v3 (MACROGEN Inc.). All RNA‐seq data were mapped using the Tophat package^[^ ^46^ ^]^ against Affymetrix Human Gene 2.0 ST arrays (902136). Remaining mRNA was used for qPCR analysis. Fold‐change was determined using the R package limma, and P‐values were Benjamini‐Hochberg (BH) adjusted. The array results are available in the Gene Expression Omnibus (GEO) database of NCBI (Accession code: GSE101126).

##### Hematoxylin and Eosin (H&E) Staining and Histopathological Examination

Male C57BL/6 mice were CLP‐operated and then intravenously administered pMNSs, free DNase‐I, or DNase‐I pMNSs (100 units) at 12 h or 24 h after CLP (*n* = 5). At 72 h post‐CLP operation, mice were euthanized. Lung specimens were removed from mice for the analysis of phenotypic changes. H&E staining was performed using a standard protocol.

##### Cytokine Levels in the Plasma of Septic Mice

Fresh serum was used for analysis of AST, ALT, BUN, creatinine, and LDH levels using biochemical kits (MyBioSource). Values were measured using an ELISA plate reader (Tecan, Austria GmbH, Austria).

##### Statistical Analysis

All the in vitro and in vivo data were analyzed via two‐tailed unpaired t‐test using the Graphpad prism 7 software, the prepared sample sizes were n ≥ 3, and the statistical significance was set at P <0.05. A more detailed information for each experiment is provided in the Figure legend. All data normalization processes were carried out according to the manufacturer's protocol. Data transformation and evaluation of outliers were not used in our study.

## Conflict of Interest

The authors declare no conflict of interest.

## Supporting information

Supporting Information
