# Bioinspired DNase‐I‐Coated Melanin‐Like Nanospheres for Modulation of Infection‐Associated NETosis Dysregulation

**DOI:** 10.1002/advs.202103748

**Published:** 2021-10-08

**Authors:** Hee Ho Park, Wooram Park, Yun Young Lee, Hyelim Kim, Hee Seung Seo, Dong Wook Choi, Ho‐Keun Kwon, Dong Hee Na, Tae‐Hyung Kim, Young Bin Choy, June Hong Ahn, Wonhwa Lee, Chun Gwon Park

*Adv. Sci*. **2020**, *7*, 2001940

DOI: 10.1002/advs.202001940

In the originally published article, there was an error in Figure 4. Please find the correct Figure 4 here:

**Figure 4:**
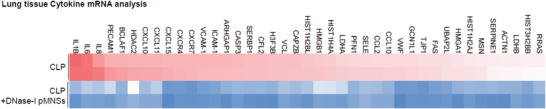
Cytokine levels in the lung tissue in CLP‐operated mouse model. Heat map of inflammation‐related genes in the lung tissue after DNase‐I pMNSs treatment (*n* = 3). CLP, Cecal ligation and perforation; pMNSs: PEG‐coated melanin‐like nanospheres.

The authors apologize for any inconvenience this may have caused.

