# Supplementary material for: Bioinspired DNase‐I‐Coated Melanin‐Like Nanospheres for Modulation of Infection‐Associated NETosis Dysregulation
Source: Adv Sci (Weinh). 2020 Oct 20;7(23):2001940. doi: 10.1002/advs.202001940 (PMC7645930; doi:10.1002/advs.202001940)
Supplement: Supplementary file 1 — Supporting Information [file ADVS-7-2001940-s001.pdf]

WILEY-VCH

© 2020 Wiley-VCH GmbH

ADVANCED SCIENCE
Open Access

Supporting Information

for *Adv. Sci.,* DOI: 10.1002/advs.202001940

Bioinspired DNase-I-coated Melanin-like Nanospheres for Modulation of Infection-associated NETosis Dysregulation

Hee Ho Park, Wooram Park, Yun Young Lee, Hyelim Kim, Hee Seung Seo, Dong Wook Choi, Ho-Keun Kwon, Dong Hee Na, Tae-Hyung Kim, Young Bin Choy, June Hong Ahn, Wonhwa Lee*, Chun Gwon Park**

Supporting Information

Bioinspired DNase-I-coated melanin-like nanospheres for modulation of infection-associated NETosis dysregulation

Hee Ho Park[‡], Wooram Park[‡], Yun Young Lee[‡], Hyelim Kim, Hee Seung Seo, Dong Wook Choi, Ho-Keun Kwon, Dong Hee Na, Tae-Hyung Kim, Young Bin Choy, June Hong Ahn[*], Wonhwa Lee[*], Chun Gwon Park[*]

Prof. H. H. P
Department of Biotechnology and Bioengineering, Kangwon National University, Chuncheon, Gangwon-do 24341, Republic of Korea

Prof. W. P
Department of Biomedical-Chemical Engineering, The Catholic University of Korea, Bucheon, 14662, Republic of Korea

Y. Y. L and Prof. Y. B. C
Department of Biomedical Engineering, Seoul National University College of Medicine, Seoul 03080, Republic of Korea

H. K

College of Pharmacy, Chungnam National University, Daejeon 34134, Republic of Korea

H. S. S

Department of Biomedical Engineering, SKKU Institute for Convergence, Sungkyunkwan University (SKKU), Suwon, 16419, Republic of Korea

Dr. D. W. C

Department of Cancer Biology, Dana-Farber Cancer Institute, Harvard Medical School, Boston, MA 02215, USA

Prof. H.-K. K

Department of Microbiology and Immunology, Yonsei University College of Medicine, Seoul 03722, Korea
Prof. D.H. N

College of Pharmacy, Chung-Ang University, Seoul 06974, Republic of Korea

Prof. T.-H. K

School of Integrative Engineering, Chung-Ang University, Seoul 06974, Republic of Korea

Prof. J. H. A

Division of Pulmonology and Allergy, Department of Internal Medicine, College of Medicine, Yeungnam University and Regional Center for Respiratory Diseases, Yeungnam University Medical Center, Daegu, 42415, Republic of Korea

E-mail: fireajh@yu.ac.kr

Dr. W. L.
Aging Research Center, Korea Research Institute of Bioscience and Biotechnology, Daejeon 34141 Republic of Korea
E-mail: bywonhwalee@gmail.com

Prof. C. G. P
Department of Biomedical Engineering, SKKU Institute for Convergence, Sungkyunkwan University (SKKU), Suwon, Republic of Korea
Biomedical Institute for Convergence at SKKU (BICS), Sungkyunkwan University, 2066 Seobu-ro, Jangan-gu, Suwon 16419, Republic of Korea

E-mail: chunpark@skku.edu

Experimental methods for Supporting Information

Measurement of binding contents of DNase-1 on the pMNSs: The binding contents of DNase-I on the pMNSs were calculated with a BCA Protein Assay Kit, performed according to the manufacturer's instructions. DNase-I pMNSs were prepared in various weight ratios of pMNSs and DNase-I (i.e. 10:2, 10:5, 10:10, and 10:20). The prepared DNase-I pMNSs were centrifuged at 17,000 rpm (27,237 × g-forces, at 4 °C) for 10 min. The supernatant was transferred to a clean tube, and a BCA Protein Assay determined the DNase-I concentration at 562 nm

Analysis of long-acting DNase-I over time under different conditions: Stability of DNase-I pMNSs was evaluated over time in PBS complemented with 10% FBS and PBS. Each sample was collected at a determined time interval (1, 3, 6, 12, 24, 36, 48, and 72 h) and analyzed by gel electrophoresis. Briefly, 1 μg of DNase-I pMNSs (weight ratio of pMNSs and DNase-I, 10:10) were incubated with 1 μg of salmon sperm DNA for 10 min at 37 ℃. Then, the activity of DNase-I was determined by gel electrophoresis.

In vivo permeability assays: CLP-operated mice were injected with pMNSs, free DNase-I, or DNase-I pMNSs intravenously. After 24 and 72 h, 1% Evans blue dye solution in normal saline was injected intravenously into each mouse. Thirty minutes later, the mice were killed, and the peritoneal exudates were collected after being washed with normal saline (5 mL) and centrifuged at 200 × g for 10 min. The absorbance of the supernatant was read at 650 nm. The vascular permeability was expressed in terms of dye (μg/mouse), which leaked into the peritoneal cavity according to a standard curve of Evans blue dye.

Table S1. Quantitative characterization of DNase-I pMNSs.

Sample	Feed (mg)		DNase-I	
	pMNSs[a]	DNase-I[b]	Binding contents (wt%)[c]	Binding efficiency (%)[d]
DNase-I pMNSs1	10	2	13.8	80.1
DNase-I pMNSs2	10	5	29.4	83.1
DNase-I pMNSs3	10	10	45.4	83.1
DNase-I pMNSs4	10	20	61.1	78.5

[a]Weight of feed pMNSs.
[b]Weight of feed DNase-I.
[c]Binding contents = (actual mass of DNase-I/actual total mass of DNase-I pMNSs) × 100, as determined by BCA assay.
[d]Binding efficiency = (actual mass of DNase-I/feed mass of DNase-I) × 100, as determined by BCA assay.

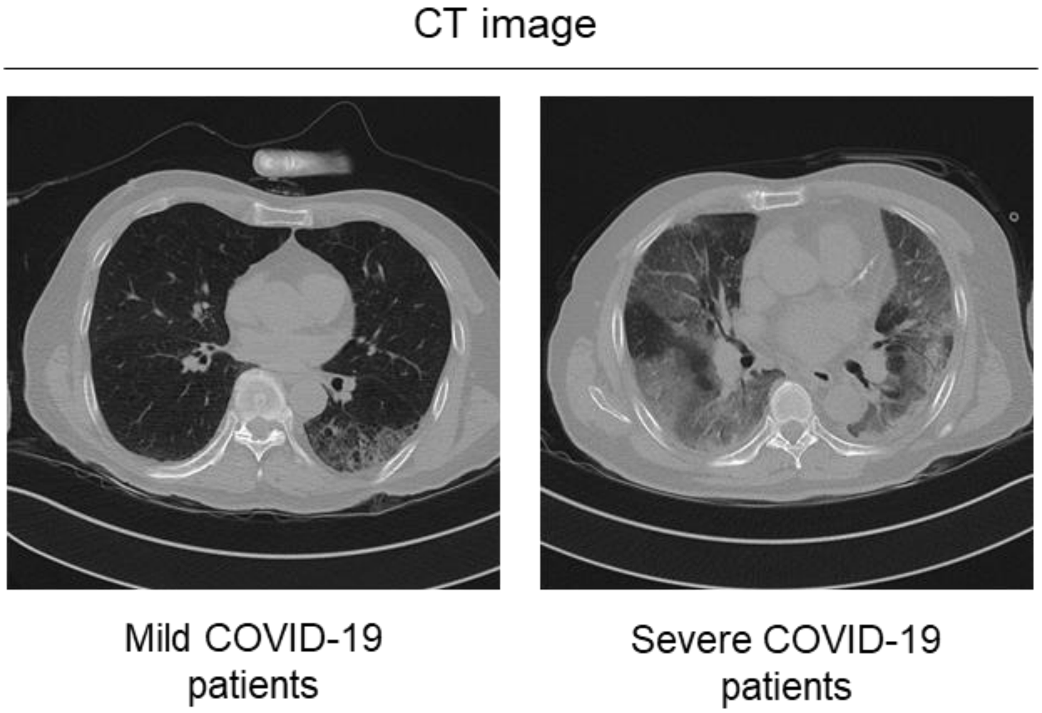

Figure S1. CT scans of mild COVID-19 (high DNase-I activity) and severe COVID-19 patients (low DNase-I activity).

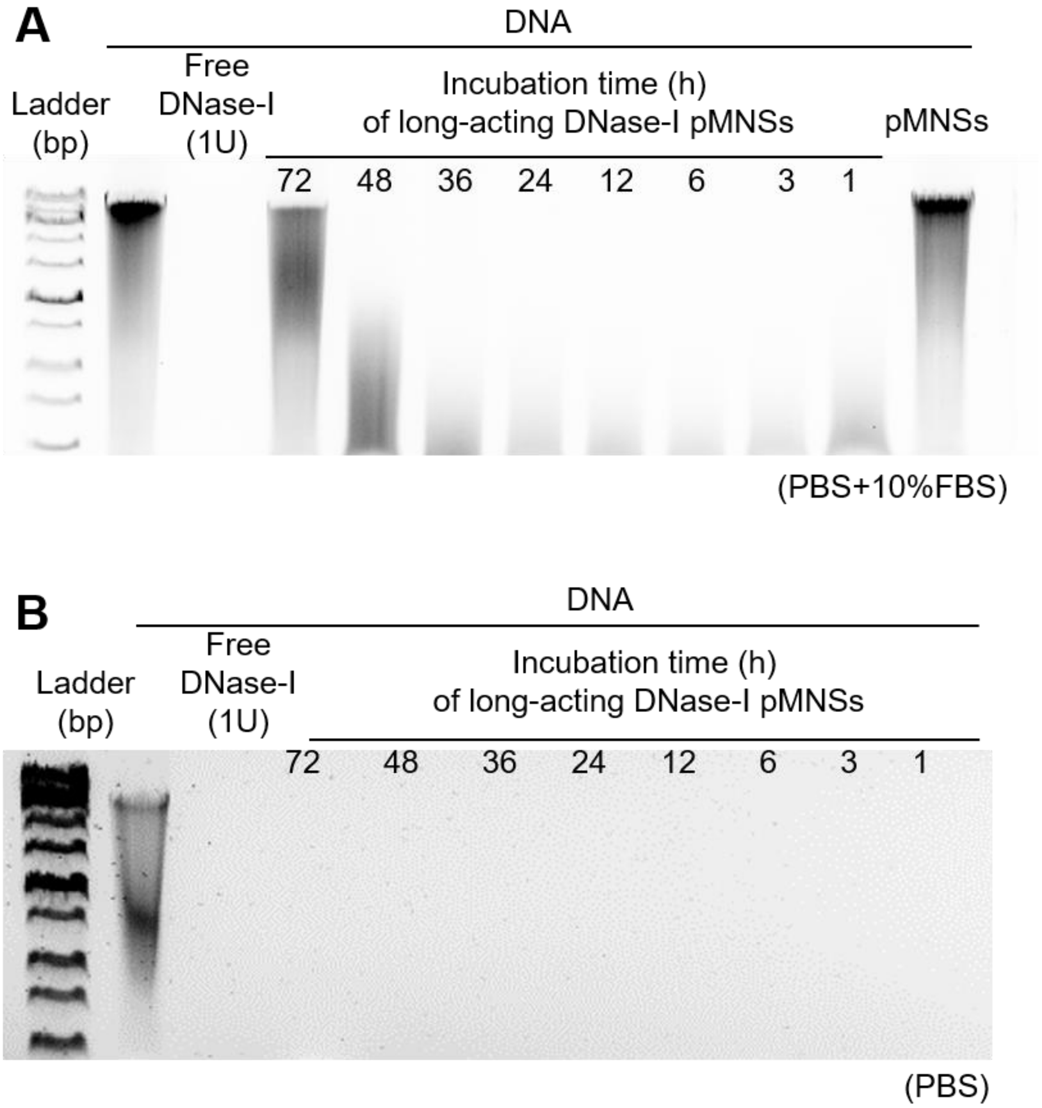

Figure S2. Analysis of long-acting DNase-I activity over time under different conditions.

(a and b) Long-acting DNase-I pMNSs (1 μg) was incubated in (a) PBS containing 10% FBS or (b) PBS only media over time, and then DNA degradation activity was measured.

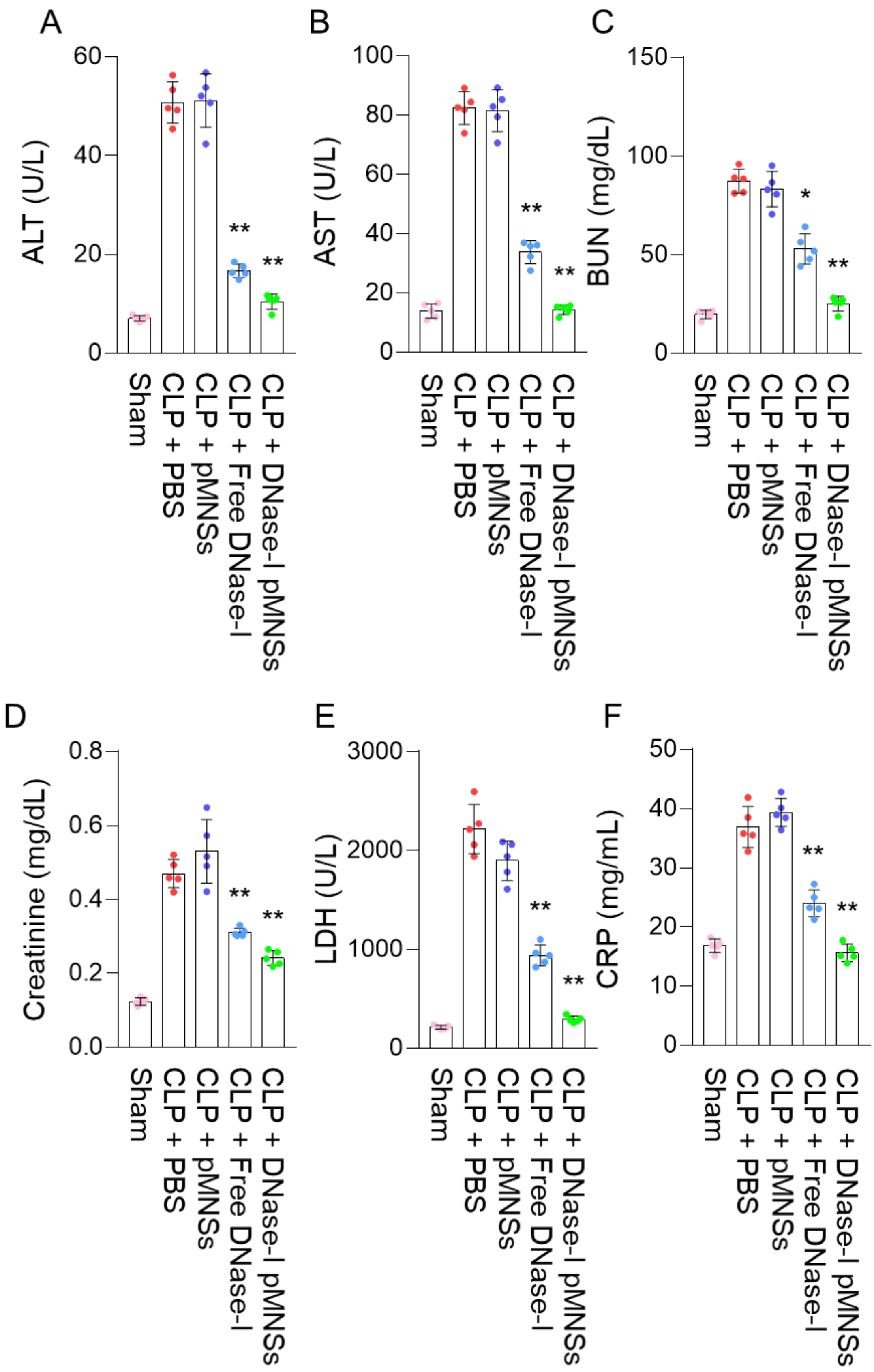
A
ALT (U/L)
60
40
20
0
**
**
B
AST (U/L)
100
80
60
40
20
0
**
**
C
BUN (mg/dL)
150
100
50
0
*
**
D
Creatinine (mg/dL)
0.8
0.6
0.4
0.2
0.0
**
**
E
LDH (U/L)
3000
2000
1000
0
**
**
F
CRP (mg/mL)
50
40
30
20
10
0
**
**
Sham
CLP + PBS
CLP + pMNSs
CLP + Free DNase-I
CLP + DNase-I pMNSs

Figure S3. Dynamic changes in septic marker levels of septic mice.

Sepsis markers (a) ALT, (b) AST, (c) BUN, (d) creatinine, (e) LDH, and (f) CRP were measured in septic mice intravenously injected with PBS, PEG-Nano (100 units), free DNase-I (100 units), or DNase-I pMNSs (100 units) at 12 and 24 h after CLP. Representative data from each group are shown (n = 5). The experiment was performed at least three times with replicates. Statistical analysis was performed using a two-tailed unpaired *t*-test. $^{*}P < 0.05$, $^{**}P < 0.01$.

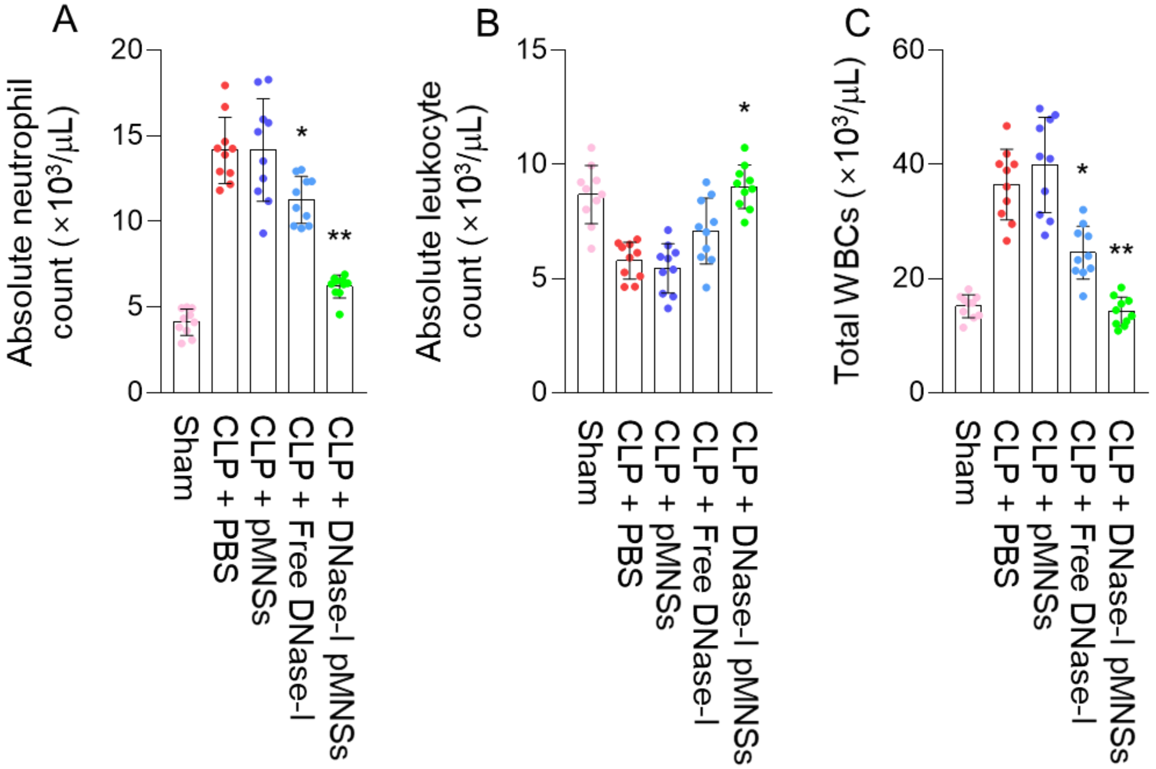

Figure S4. Quantified mean white blood cells in septic mice. Cell counts (cells x 10^3 /μL) are presented as (a) absolute neutrophil count, (b) absolute leukocyte count, and (c) total white blood cell (WBC) count in each group (n=10). Statistical analysis was performed using a two-tailed unpaired *t*-test. $^{*}P < 0.05$, $^{**}P < 0.01$.

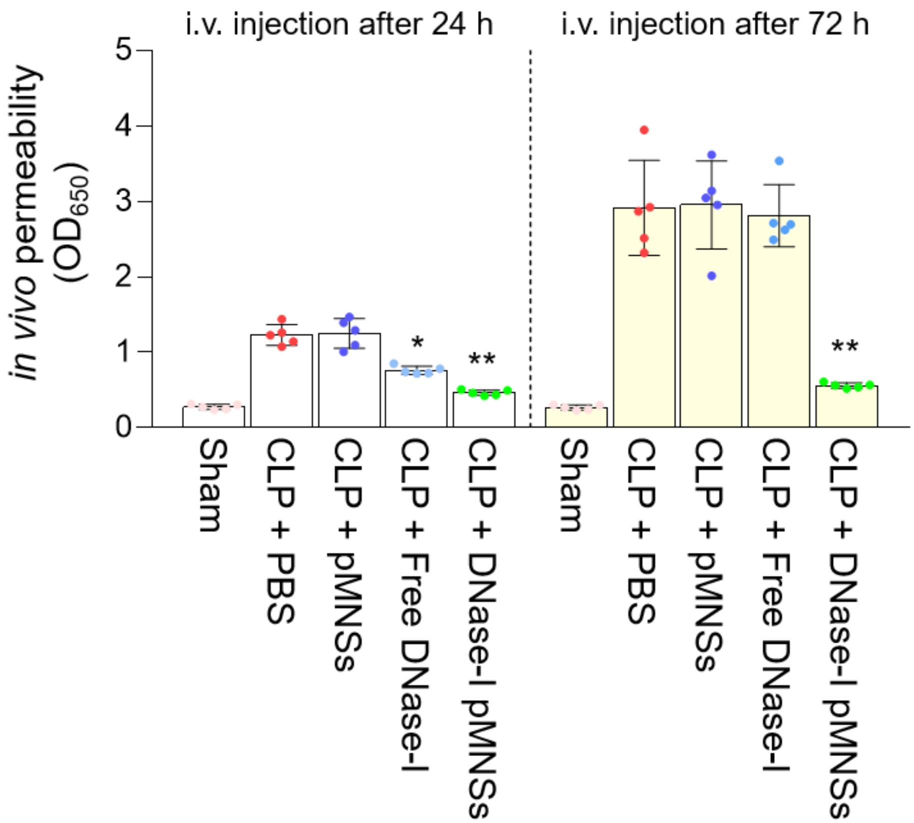

Figure S5. Recovery of vascular barrier integrity after the treatment of DNase-I pMNSs. In vivo permeability was evaluated at 24 and 72 h after administration of PBS, PEG-Nano (100 units), free DNase-I (100 units), or DNase-I pMNSs (100 units) (n=5/each group). *p<0.05, **p<0.01.

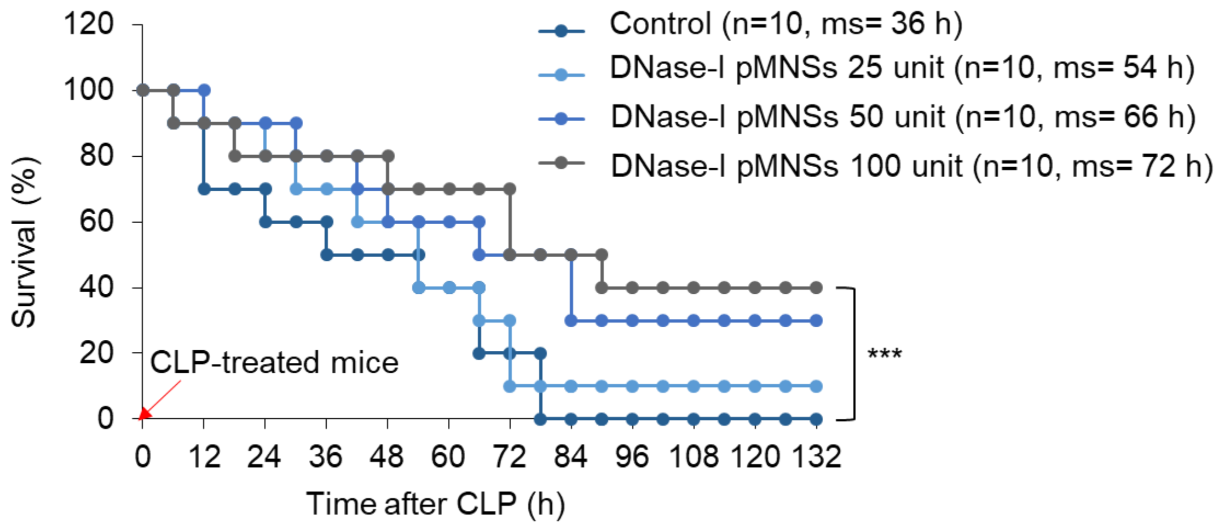

Figure S6. The survival rate of CLP-operated mice models after the treatment of DNase-I pMNSs. Kaplan–Meier curves of CLP mice administered with PBS or DNase-I pMNSs (25, 50, or 100 units) at 12 and 24 h after CLP (n=10/each group). ***p<0.005.
